# *Lacticaseibacillus rhamnosus* Strains for Alleviation of Irritable Bowel Disease and Chronic Fatigue Syndrome

**DOI:** 10.3390/microorganisms12061081

**Published:** 2024-05-27

**Authors:** Liang Zhang, Xue Ni, Minzhi Jiang, Mengxuan Du, Shuwen Zhang, He Jiang, Chang Liu, Shuangjiang Liu

**Affiliations:** 1 State Key Laboratory of Microbial Technology, Shandong University, Qingdao 266237, China; 202190900070@sdu.edu.cn (L.Z.); nixue1140317984@foxmail.com (X.N.); jiangminzhi2006@126.com (M.J.); dumengxuan2014@126.com (M.D.); 202000141061@mail.sdu.edu.cn (S.Z.); jiangh@sdu.edu.cn (H.J.); liu.c@sdu.edu.cn (C.L.); 2 State Key Laboratory of Microbial Resources, Institute of Microbiology, Chinese Academy of Sciences, Beijing 100101, China

**Keywords:** *Lacticaseibacillus rhamnosus*, pan- and core-genomes, metabolic and genetic diversity, chronic fatigue syndrome (CFS), irritable bowel syndrome (IBS), strain-specific probiotics

## Abstract

*Lacticaseibacillus rhamnosus* is applied as a probiotic to alleviate various metabolic, gastrointestinal, and psychological symptoms and diseases, and its probiotic effectiveness is strain-specific. In this study, we obtained 21 strains of *Ls. rhamnosus*, and their genomes were sequenced. We defined the pan- and core-genomes of *Ls. rhamnosus*. Phenotypes such as the assimilation of carbohydrates and antibiotic resistance were experimentally characterized and associated with genome annotations. Nine strains were selected and tested for growth rates, tolerance to acidity/alkalinity and bile acids, the production of short-chain fatty acids, and competition with pathogenic microbes. Strains WL11 and WL17 were targeted as potential probiotics and were applied in mouse model tests for the alleviation of chronic fatigue syndrome (CFS) and irritable bowel syndrome (IBS). The results showed that WL11 and WL17 effectively alleviated slow body weight gain, anxiety, poor memory, and cognitive impairment in CFS mouse models. They also reduced the expression of pro-inflammatory factors, such as TNF-α and IL-6, and alleviated intestinal peristalsis, visceral hypersensitivity, and anxiety-like behavior in IBS mouse models. This study reports new *Ls. rhamnosus* strain resources and their effect on alleviation of both IBS and CFS symptoms with mouse models; the probiotic functions of those strains in human patients remain to be further tested.

## 1. Introduction

Chronic fatigue syndrome (CFS) and irritable bowel syndrome (IBS) are complex diseases and are distributed worldwide, and the estimated prevalence rates are 3–20% (CFS) and 5–10% (IBS) of the world population [1,2,3,4,5]. CFS patients suffer from excessive fatigue, post-exertional malaise, cognitive dysfunction, and immune alterations [6,7], and they often have gastrointestinal disorders as common symptoms. More than 58% of CFS patients meet the criteria for IBS [8,9]. IBS patients suffer from functional gastrointestinal disorders, poor life quality, and poor social function [10]. CFS and IBS have become a major challenge for medical care, social development, and economic development [11].

Probiotic bacteria effectively relieve the symptoms of CFS and IBS in clinical studies as well as in medical practice [12,13,14]. *Lacticaseibacillus rhamnosus* is one of the most used probiotic species. Studies show that *Ls. rhamnosus* strains such as LGG and HN001 were able to up-regulate the expression of SERT proteins, enhance the intestinal barrier, modulate the immune system of IBS patients [15,16,17]. The effects of *Ls. rhamnosus* on relieving IBS symptoms are quite strain-specific, and a case was reported where the *Ls. rhamnosus* strain R0011 was ineffective in the alleviation of gastrointestinal disorders [18]. Many probiotic bacteria genetically enable themselves to compete with pathogens, modulate intestinal environments and host nutrients, regulate the host immune system and protect the host intestinal mucous barrier, and produce health-promoting metabolites such as short-chain fatty acids (SCFAs) [19,20,21]. So far, there have been no reports on the diversity of *Ls. rhamnosus* strains that alleviate both CFS and IBS.

In this study, 21 strains of *Ls. rhamnosus* were obtained from the intestines of healthy volunteers. The *Ls. rhamnosus* genomes were sequenced, and the strains were phenotypically characterized and correlated with genome annotations. Two strains (namely WL11 and WL17 = GOLDGUT-L818) were targeted for probiotic use and effectively alleviated CFS and IBS symptoms with mouse models.

## 2. Materials and Methods

### 2.1. Fecal Sample Collection, Bacterial Isolation, and Identification

Fecal samples from healthy Chinese volunteers were collected and suspended in PBS buffer with 0.1% L-cysteine, large insoluble particles in the suspension were removed using a cell strainer, diluted (10^−2^, 10^−4^, 10^−6^, 10^−8^), and 200 μL of each dilution was plated onto modified peptone yeast extract glucose (PYG) and de Man–Rogosa–Sharpe (MRS) agar plates (Qingdao Hope Bio-Technology Co., Ltd., Qingdao, China, containing L-cysteine at 0.05%). The plates were incubated at 37 °C under an atmosphere of 85% of N_2_, 10% of CO_2_, and 5% of H_2_. Colonies on the agar plates were picked after incubating for 2, 3, and 4 days. The picked colonies were then inoculated into Hungate tubes, which were incubated at 37 °C for 2 days [22]. Strain P001 was isolated from a commercial product and was used as a reference for test. The cultivated bacterial strains were identified with 16S rRNA gene sequences and phenotypic characterization (BIOLOG tests). The 16S rRNA genes were PCR-amplified and the preparations of DNA templates were based on Liu et al. [23].

### 2.2. Genome Sequencing, Phylogenomic Tree Construction, and Annotations for Antibiotic Resistance, Carbohydrate Utilization, and Virulence Factors

The cells of the *Ls. rhamnosus* strains were collected by centrifugation at 5180× *g* for 5 min. DNA sequencing and genome assembly of *Ls. rhamnosus* strains were carried out by Magigene Tech Co., Ltd. (Shenzhen, China), using a protocol of combining the Illumina HiSeq2000 (San Diego, CA, USA) and Nanopore (Oxford Nanopore Technologies, Oxford, UK) sequencing platforms. Genomes with integrity greater than 95% were annotated using Prokka (version 1.14.6) [24]. The output files of Prokka were sent to Roary (version 3.13.0) [25] for analysis of the pan-genome and core-genome with the default parameters. Phylogenomic identification was performed by an ANI analysis using homologous average nucleotide identity (OrthoANI) [26]. A core-genome-based neighbor-joining unrooted a phylogenomic tree using Roary [25] and FastTree [27], and the phylogenomic tree was visualized using the iTOL web tool [28]. The antibiotic resistance genes were identified with Comprehensive Antibiotic Resistance Database (CARD) web tool [29] using an identity threshold of 50%. The carbohydrate utilization genes were annotated using the carbohydrate-active enzymes (CAZymes) [30]. Virulence factors were searched against the VFDB database [31] using an identity and coverage threshold of 20%. The genomic data for the strains in this study were deposited in the National Microbiology Data Center (NMDC) with accession numbers NMDC10018728 (https://nmdc.cn/resource/genomics/project/detail/NMDC10018728, accessed on 16 May 2024).

### 2.3. Determination of Generation Time and Tests for Acidity and Alkalinity Tolerance

Fresh bacterial broths were inoculated into a MRS medium and incubated anaerobically at 37 °C. Bacterial growth was measured using a spectrophotometer at a wavelength of 600 nm (OD_600_) every 2 h. The generation time was calculated with the equation *n* = (log Pt − log P0)/log2, where P0 and Pt were the initial OD_600_ and the end OD_600_ of the bacterial cultures, respectively [32]. 

The tolerance to acidity and alkalinity was tested in a MRS medium and PBS buffer (pH 3, 3.5, 4, 7, 9, and 10). The cells were cultured anaerobically at 37 °C for 24 h, and were collected by centrifugation, resuspended with PBS buffer, and incubated at 37 °C for 4 h. The survival rate was calculated using the dilution coating method, according to the following equation:

$$\left(\%\right)\;\mathrm{survival}=\frac{\log\;\mathrm{CFU}\;\mathrm{cells}\;\mathrm{survived}}{\log\;\mathrm{initial}\;\mathrm{cells}\;\mathrm{inoculated}}\times 100\%$$

### 2.4. Carbohydrate Utilization, Production of Short-Chain Fatty Acids and Organic Acids, and Antibiotic Susceptibility Assays

The ability of *Ls. rhamnosus* to metabolize different carbohydrates was investigated using 96-well BIOLOG ANI microplates (BIOLOG Inc., Hayward, CA, USA) with 95 different carbohydrate substrates and a negative control. According to the manufacturer’s directions, a bacterial suspension was prepared by a collection of freshly grown cells from the MRS liquid medium and washed three times with PBS. After inoculation, the microplates were incubated anaerobically at 37 °C for 24 h. Growth and carbohydrates were evaluated by following the changes in absorbance at wavelengths of 590 nm and 750 nm of each well of the microplates.

Short-chain fatty acids (SCFAs, including acetic, propionic, isobutyric, butyric, and isovaleric acids) and organic acids (including lactic, succinic, and malic acids) were determined with GC/MS equipped with a DB-5MS capillary column [33]. The cells were grown in the MRS broth. The supernatant of the *Ls. rhamnosus* fermentation broth was extracted 3 times with an equal volume of ethyl acetate for SCFAs analysis, and organic acids were determined by derivatization. The volume of 300–400 μL organic phase was loaded for GC/MS analysis.

The test of antibiotic susceptibility was performed following the disc diffusion method described by Halder and Mandal [34]. *Ls. rhamnosus* strains were inoculated onto MRS agar, and then antibiotic discs with various antibiotics were introduced. The diameter of the resultant inhibition zones was measured after anaerobic incubation at 37 °C for 24 h.

### 2.5. Inhibition of the Growth of Causative Pathogenic Indicator Microbes

The inhibition of *Ls. rhamnosus* strains against causative pathogenic indicators was determined using the agar well diffusion method [35]. The causative pathogenic indicators, including *Bacillus subtilis* 1.8, *Staphylococcus aureus* ATCC 6538, *Pseudomonas aeruginosa* ATCC 27853, *Escherichia coli* ATCC 25922, and *Candida albicans* 1.321 from the Shandong University Strain Collection Center, were cultured in LB or TSA liquid media, and the cell concentration was adjusted to 1 × 10^7^ CFU/mL. LB and TSA solid media were perforated (5 mm) in sterile conditions and coated with 200 μL indicator cell concentrates. A total of 100 μL of cell fermentation supernatant of the tested strain was added into the well, and sterile distilled water was used in the control well. The culture dish was cultured at 37 °C for 12 h, and the diameter of the inhibition zone was recorded after culture. Each strain was repeated three times, and the inhibition ability was expressed by the size of the inhibition zone.

### 2.6. Experimental Design of CFS and IBS Model Animals

The animal studies have complied with the requirements of the Helsinki Declaration and was approved by the Ethics Committee of Shandong University for the care and use of laboratory animals [Approved No. SYDWLL-2023-063]. Female SPF C57BL/6J mice, aged 5–6 weeks, were purchased from Beijing Vital River Laboratory Animal Technology Co., Ltd. (Beijing, China) and housed under controlled temperature (24 ± 2 °C), relative humidity (60–80%), and a 12 h light/dark cycle in specific pathogen-free (SPF) animal rooms; these conditions were maintained. All the mice were fed sterile water and a normal chow diet ad libitum. 

The chronic fatigue syndrome (CFS) mouse model was established using a modified method as previously described [36,37]. All mice adapted to the laboratory environment for one week and according to the experimental requirements were randomly selected and divided into groups (6 mice per group). Before the start of modeling, each group of mice was placed in the interference box of the sleep deprivation device for adaptation, for 7 consecutive days and 3 h per day. In the first week of the experiment, *Ls. Rhamnosus*-treated group mice received a daily intake of *Ls. rhamnosus* strain (2 × 10^9^ CFU/day/piece). During weeks 2–11 of the experiment, all mice in the model group and the *Ls. Rhamnosus*-treated group were subjected to sleep deprivation, with 23 h of sleep deprivation and light interference per day and 1 h of rest.

Male SPF C57BL/6J mice, aged 5–6 weeks, were fed under the same conditions as the CFS modeling experiment. The mice adapted to the laboratory environment over one week and, according to the experimental requirements, were randomly selected and divided into groups (6 mice per group). Repeated water avoidance stress (WAS) procedures were conducted as previously described to establish the IBS mouse model [38]. All the mice were subjected to WAS stimulation using a WAS device, which consisted of a 45 × 25 × 25 cm box with a 10 × 8 × 8 cm dry platform at the bottom of the box. The box was filled with water, and the water level was 1 cm lower than the platform. Except for the control group, the mice were placed on a dry platform for 1 h per day. The control group was placed on a dry platform in a water-free container. For the *Ls. rhamnosus*-treated group, the mice took dosages of *Ls. rhamnosus* (2 × 10^9^ CFU per mouse per day) for five consecutive days and were subsequently subjected to WAS treatment from day 6 till day 16. The male mice in model and control groups were used as the normal control treated with a vehicle. 

Biochemical parameters of serum, such as the levels of TNF-α, IL-6, and MDA in the mouse serum were analyzed using enzyme-linked immunosorbent assays (ELISAs) following the manufacturer’s instructions (Boshen, Zhongshan, China).

### 2.7. Visceral Sensitivity Assessment

Colorectal distension tests were performed the day after the last WAS session to determine the visceral sensitivity of mice. Mice were anesthetized with an intraperitoneal injection of urethane, and a paraffin oil-coated urinary catheter was inserted into the colorectum of the mice. The tail end was 1 cm away from the anal opening, the catheter was fixed at the tail of the mouse with tape, and the mouse took 20 min to adapt after waking up. Colorectal dilation was performed using gas pressure values of 0.05, 0.1, 0.15, and 0.2 mL. The target pressure was reached within 3 s, and maintained for 20 s, with a stimulation interval of 4 min. Under unknown pressure, the abdominal wall reflex retraction in the mice was observed and scores were recorded. The experiment was conducted in triplicate. The AWR score was used to evaluate the visceral sensitivity of mice. Scoring criteria were as follows: 0 points, no obvious response to distension; 1 point, brief head movement followed by immobility; 2 points, contraction of abdominal muscles; 3 points, lifting of abdomen; 4 points, body arching and lifting of polar structures.

### 2.8. Tests with Elevated Plus-Maze (EPM), Rotating Rod, and Y-Maze

The mice were videotaped for 6 min after placement on the center platform in the elevated plus-maze located in a dark–light and quiet room. Behavioral observations were captured using a video camera and an ethological analysis system, including total arm entries, open arm entries, time spent on the open arms, and the percentage of time spent on the open arms relative to the total time.

For the rotating rod test, the mice were trained on a rotating rod for 3 consecutive days before the formal experiment and rotated at a constant speed of 30 rpm. In the formal experiment, the mouse rotated at an initial speed of 1 rpm which was gradually increased to 30 rpm, recording the latency of the mice falling off within 5 min.

The cognitive function test of the mice was performed using the Y-maze. The three arms were randomly assigned the start arm, novel arm, and other arms. In the training phase of the test, occluding the novel arm, the mice were placed in the start arm to explore for 10 min freely. After 2 h, the mice were returned to the same start arm and permitted to move freely for 5 min. The time spent in the novel arm and the distance of motion in the novel arm were recorded for the function of spatial recognition memory.

### 2.9. Statistical Analysis

All the data were expressed as the standard error of the mean (SEM). The statistical significance of the difference was evaluated using one-way analysis of variance (ANOVA) followed by Tukey’s post hoc test. *p*-values less than 0.05 were considered statistically significant. All statistical analyses, the box–whisker plots, and the bar charts were performed or created using GraphPad Prism v8 (GraphPad Software, La Jolla, CA, USA).

## 3. Results and Discussion

### 3.1. Ls. rhamnosus Strain Diversity and Their Genomic Features

**Figure 1:**
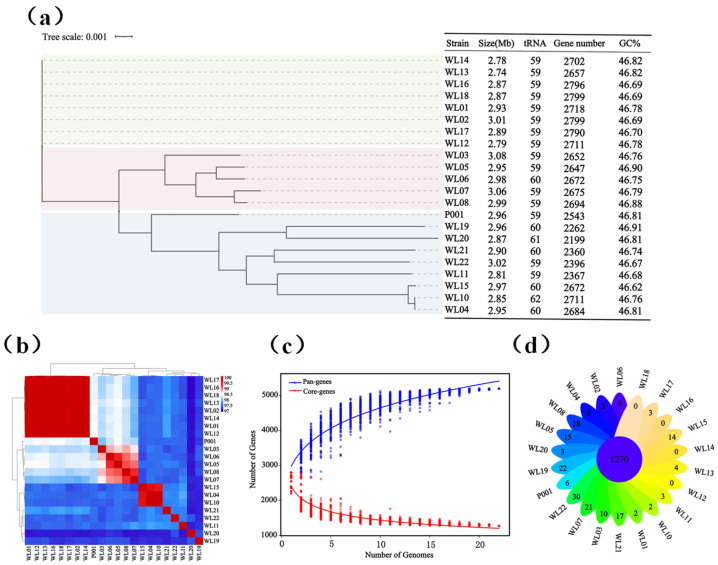
The general genomic features and phylogeny (**a**), Average Nucleotide Identity (ANI) (**b**), and pan- and core-genomes (**c**,**d**) of 22 *Ls. rhamnosus* strains. The phylogenetic tree was constructed with the neighbor-joining method based on core gene alignment. The pan- and core-genome analysis shows the growing pan-gene numbers as genome numbers increase (**c**) and 1270 core genes (**d**) of 22 *Ls. rhamnosus* strains. The Venn diagram displays the unique and core genes.

Twenty-one strains that phylogenetically closely clustered with *Ls. rhamnosus* strain ATCC 7469^T^ were obtained, using MRS and PYG culture media and their taxonomies were identified according to 16S rRNA genes and genomes. Their 16S rRNA gene sequences showed high identities (99.1–99.8%) to *Ls. rhamnosus* strain ATCC 7469^T^. The genome sizes of the 21 strains of *Ls. rhamnosus* ranged from 2.74 Mb to 3.08 Mb, with 59–62 copies of tRNA genes. Their GC contents were quite close, ranging from 46.62% to 46.91%. Based on the alignment of the core genes from the 21 strains, *Ls. rhamnosus* mainly existed in three evolutionary branches (Figure 1a). We further compared the ANI values of the 21 genomes of *Ls. rhamnosus* in order to explore any genomic differences. The results showed that their ANI values ranged from 97% to 99% (Figure 1b), which was consistent with the species cutoff value (>95%) [39,40] and indicated that these 21 strains belonged to the species *Ls. rhamnosus*.

With the 21 newly sequenced genomes and the genome of strain P001 (from probiotic product), we performed pan- and core-genome analysis for *Ls. rhamnosus*. The results showed that *Ls. rhamnosus* had a pan-genome of 5197 orthologs and a core-genome of 1270 orthologs (accounting for 24.4% of the pan-genome). There were 3908 accessory genes, accounting for 73.3% of the pan-genome. As shown in Figure 1c, the size of the pan-genome increased with the number of strains, indicating that the *Ls. rhamnosus* has an open pan-genome, which suggests *Ls. rhamnosus* have strain and genetic diversities. The analysis of the pan-genome in *Ls. rhamnosus* strains revealed a variation in the numbers of unique genes within these strains, ranging from 0 to 30 (Figure 1d).

*Ls. rhamnosus* exists in a variety of ecological niches, including the intestinal tract, dairy products, soil, etc., and is widely used as a fermentation agent in the food industry and as probiotics for alleviating metabolic syndrome, improving immunity, and reducing inflammatory responses in colitis mice [41]. This current study reported new resources of 21 strains of *Ls. rhamnosus* and their genomes, defined the pan- and core-genomes of *Ls. rhamnosus*, and significantly expanded the strain and genome resources [42].

### 3.2. Genome Annotation and Experimental Determination of Carbohydrate Utilization, Resistance to Antibiotics, and Prediction of Virulence Factors 

**Figure 2:**
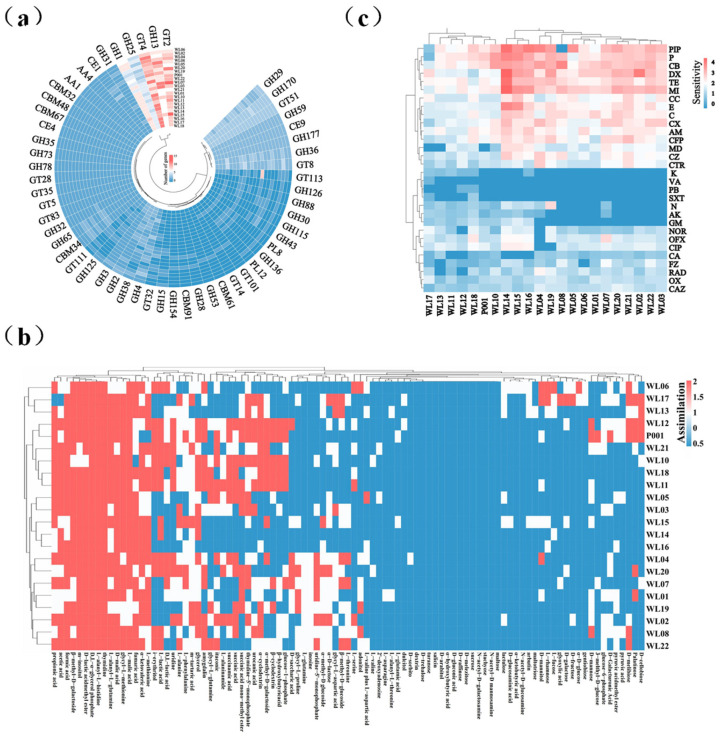
Genome annotations and experimental tests for carbohydrate utilization enzymes (**a**,**c**) and antibiotic resistance sensitivity test (**b**). (**a**) Genome annotations; (**b**) BIOLOG test for carbon source assimilation; (**c**) antibiotic resistance sensitivity test. The *Ls. rhamnosus* strain P001 was analyzed and tested in parallel for reference. Abbreviations: P: penicillin; OX: oxacillin; AM: ampicillin; CB: carbenicilli; PIP: piperacillin; CA: cephalexin; CZ: cefazolin; RAD: cephradine; CXM: cefuroxime; CAZ: ceftazidime; CTR: ceftriaxone; CFP: cefperazone; AK: amikacin; GM: gentamicin; K: kanamycin; N: neomycin; TE: tetracycline; DX: doxycycline; MI: minocycline; E: erythromycin; MD: medemcyin; NOR: norfloxacin; OFX: ofloxacin; CIP: ciprofloxacin; VA: vancomycin; PB: polymyxinB; CC: clindamyci; SXT: trimethoprim-sulfamethoxazole; FZ: furazolidone; C: chloramphenicol. BIOLOG test for carbon source assimilation with *Ls. rhamnosus* strains (**c**). The *Ls. rhamnosus* strain P001 was analyzed in parallel for reference.

In order to understand the carbohydrate utilization ability of *Ls. rhamnosus* strains, we annotated the 21 newly sequenced genomes using the CAZy database, and experimentally determined their carbon source assimilation with BIOLOG plates. The pan-genome of *Ls. rhamnosus* covered genes of 28 glycoside hydrolases (GHs), 13 glycosyltransferases (GTs), 3 carbohydrate esterases (CEs), 6 carbohydrate binding modules (CBMs), 2 polysaccharide lyases (PLs), and 2 auxiliary activities (AAs). The most abundant enzymes were the members from the GHs family, and GH1, GH13, GH25 and GH170 were the most numerous ones in the GHs family; The second most abundant enzymes were the GTs family. We found that 54 genes encoding carbohydrate-active enzymes existed in all 21 genomes, such as GT4 (sucrose synthase), GH1 (β-glucosidase), CE1 (feruloyl esterase), and GH13 (α-amylase). Based on the distribution of the carbohydrate-active enzymes genes, the 21 strains were divided into three clusters (Figure 2a). BIOLOG plates were used to test carbon source utilization by the 21 strains. The results showed that the strains containing the GH13 and GH32 genes were able to assimilate D-fructose, indicating that the genotype was consistent with the phenotypic results (Figure 2b). However, inconsistency occurred between genotype and phenotype, for example, some strains utilized D-trehalose, but there were no related genes annotated, and others such as strains WL09 and WL10 did not utilize lactose although their genomes had the lactase gene (GH2). We also evaluated the capacity of various *Ls. rhamnosus* strains to utilize amino acids and organic acids, and the results revealed differences in their utilization efficiencies (Figure 2b), indicating there was metabolic diversity of *Ls. rhamnosus* strains. 

Sensitivity to clinic antibiotics and production of virulence factors are mostly relevant for the evaluation of probiotic strains. We annotated the resistance and virulence genes of the 21 newly sequenced genomes of *Ls. rhamnosus*. The results showed that there were genes for fluoroquinolone, fusidane, and lincosamide resistance in all 21 strains, but there was no gene for sulfonamide resistance (Supplementary Materials, Figure S1a). Experimental results showed that the *Ls. rhamnosus* strains were sensitive to ciprofloxacin and clindamycin (Figure 2c). The differences in the number of genes and in their resistance to the tested antibiotics indicated that *Ls. rhamnosus* strains were diversified in their resistance to antibiotics. Virulence factors (VFs) are important for evaluating the pathogenicity of bacteria. The virulence factors in the genomes of *Ls. rhamnosus* were mainly related to immune modulation and adherence (Supplementary Materials, Figure S1b), and their similarities to those of pathogenic bacteria were low. As previously demonstrated [43], those annotated VFs of the newly obtained 21 strains were most likely non-pathogenic to host health, and a further assessment on a couple of the newly isolated strains by a third party indicated that they are safe.

### 3.3. Growth Rates, Tolerance to Low/High pH, Bile Acids and Production of Short-Chain Fatty Acids by Nine Selected Strains

Based on the above results, such as the phylogenomic tree and carbohydrate utilization ability. We selected nine strains that were potentially suitable as probiotics. The nine strains were subjected to further characterization of their growth rates (generation time), tolerances to extreme pH and bile acids, and production of SCFAs. The growth rates of *Ls. rhamnosus* strains varied, with generation times ranging from 112 to 159 min (Supplementary Materials, Table S1). Among the nine strains tested, the fast-growing ones were strains WL11 and WL17, with generation times of about 112 min. The strain WL16 was the slowest one, with a generation time of 159 min. Generation time is a direct indicator of bacterial growth rate, which affects the cost of production and the effectiveness of probiotics [44]. Fast-growing lactic bacteria showed advantages over slow-growing ones in their colonization of the mouse intestines [45]. Survival under gastrointestinal tract conditions and the colonization of the intestine are necessary for probiotics to exert benefits on the host’s health [46,47]. The nine strains survived at pH 3.5, but only strains WL11, WL17, and WL18 survived at pH 3.0 with survival rates >90%. Compared to the low pH, all nine strains showed excellent tolerance to higher pH, and survival rates were >95% even at pH 10 (Supplementary Materials, Table S1). We also determined their tolerance to bile acids, and the results showed the strains did not tolerate bile acids.

**Figure 3:**
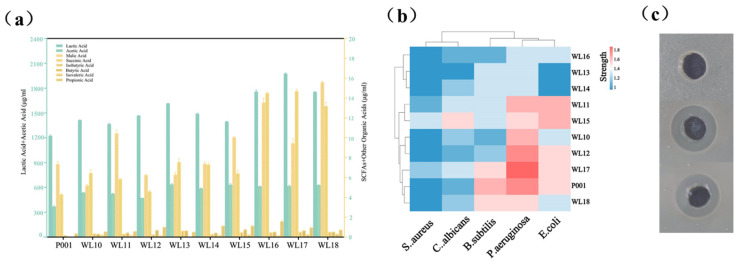
Production of lactic acid and SCFAs/other organic acids (**a**) and inhibition on growth of 4 microbial indicators (**b**) by 9 strains of *Ls. rhamnosus*. Panel (**c**) shows a representative picture of the competition/inhibition test.

We quantified the nine strains production of SCFAs and other organic acids (lactic, succinic, and malic acids), and evaluated their inhibition of pathogenic microbes. As expected, acetic and lactic acids were the main acids, and strain WL17 was the most productive for acetic and lactic acids, with productions of 616 and 1975 mg/L, respectively (Figure 3a). We did not determine the performance of L- or D-lactic acid. According to genome annotation, there were homologs to D-lactic acid dehydrogenase. Thus, strains WL17 and others possibly produced mixtures of L- and D-lactic acids. The production of SCFAs such as propionic, butyric, isobutyric, and isovaleric acids was relatively low, ranging from 0 to 1.59 μg/mL. The production of succinic and malic acids ranged from 4.3 to 15.6 μg/mL. As shown in Figure 3b,c, all nine *Ls. rhamnosus* strains were able to inhibit the growth of indicator pathogenic microbes. Strains WL11 and WL15 were able to produce the strongest inhibition against *E. coli* with inhibition zones of 1.6 cm. The maximum inhibition against *C. albicans* appeared with WL15, and its inhibited zone is 1.5 cm. The maximum inhibition against *P. aeruginosa* reached 1.7 cm in diameter by strain WL17. For *Staphylococcus aureus*, the effective strains WL15 and WL17 formed inhibition zones of 1.3 cm and 1.2 cm in diameter, respectively. The inhibitory ability of probiotics against opportunistic pathogens is one of their important characteristics. Lactic acid bacteria can produce a variety of antibacterial metabolites, such as hydrogen peroxide, organic acid, bacteriocins, and diacetyl [48,49]. The mechanisms via which the strains WL11 and WL17 inhibit the growth of pathogenic microbes remains to be investigated. 

Based on the above results, we selected WL11 and WL17 (= GOLDGUT-L818) for further experiments on their probiotic effects on mouse models of IBS and CFS.

### 3.4. Strains WL11 and WL17 Alleviate Symptoms in IBS Mouse Model

**Figure 4:**
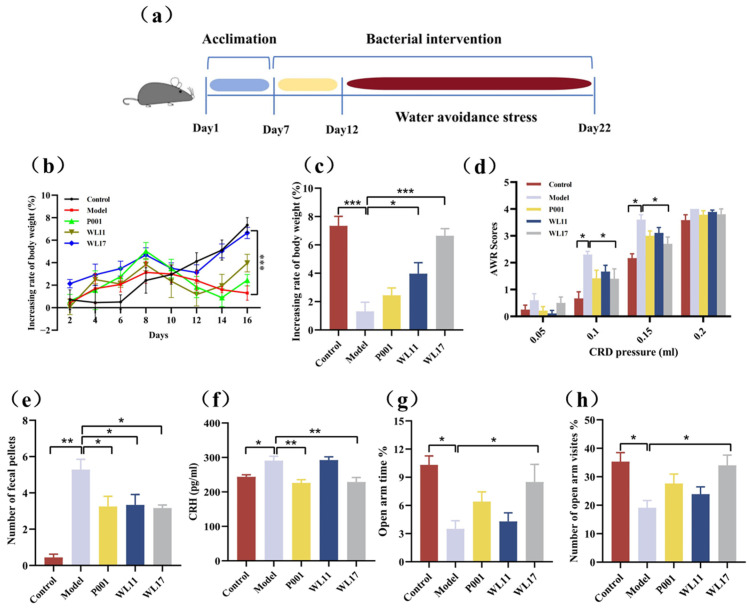
*Ls. rhamnosus* strains WL11 and WL17 alleviated symptoms of WAS-induced IBS mice (n = 6/group). Abbreviations: WAS: water avoidance stress; CRD: colorectal distension; AWR: abdominal withdrawal reflex; CRH: corticotropin-releasing hormone. Panels: Experimental process diagram (**a**); Changes in body weight (**b**) and the percentage of weight gain on the last day (**c**); Abdominal withdrawal reflex scores in response to CRD (**d**); Number of fecal pellets found in containers during WAS on the last day (**e**); Effect of different strains on the hypothalamic–pituitary–adrenal axis index of CRH (**f**) in mouse serum (data of day 16); Percentage of time spent in the open arms of the EPM (**g**); Percentage of entries into open arms (**h**). Statistics: * *p* < 0.05, ** *p* < 0.01, *** *p* < 0.001. Strain P001 was from Wonderlab storage and was evaluated in parallel in all tests.

We evaluated the beneficial effects of *Ls. rhamnosus* strains WL11 and WL17 on WAS-induced IBS in mice (Figure 4). During the 16 days of treatment, the model group of mice (without any intake of *Ls. rhamnosus* strains) gained body weight slowly, and the experimental group of mice (with intake of *Ls. rhamnosus* strain WL11 or WL17) restored body weight gain (Figure 4b). At the end of experiments, the experimental group of mice treated with strain WL17 had a similar body weight as that of the control group of mice (with not stress from WAS) (Figure 4c). The CRD tests were used to evaluate visceral hypersensitivity in IBS model mice; compared with the control group, the model mice group showed visceral hypersensitivity. The strain WL17 significantly attenuated the increased the AWR scores of the stressed mice with 0.1 and 0.15 mL of air pressure at the end of the experiment (*p* < 0.05) (Figure 4d). All three groups of the experimental mice that were treated with either strain P001, WL11, or WL17 significantly decreased fecal pellet output numbers compared with those in the model group (Figure 4e). 

To explore the effect of *Ls. rhamnosus* on alleviating anxiety-like behaviors in IBS mice, EPM tests were applied for all the mice. As shown in Figure 4f,g, the percentages of time spent exploring the open arm and the percentage of the number of entries to open arms were significantly higher in the model group than in the control group. The results demonstrated that WAS profoundly increased anxiety-like behaviors in mice. Nevertheless, the percentage of time spent exploring open arms and the percentage of the number of entries to open arms were significantly increased by WL17 supplementation. Based on the results of the EPM, we confirmed that strain WL17 could alleviate anxiety-like behaviors in mice. We also measured the serum CRH. The results showed significant differences in serum CRH levels between the control and model groups, and strains WL17 and P001 prevented the elevation of serum CRH levels in IBS mice (Figure 4h). This suggests that the improvement in IBS symptoms by strain WL17 may be related to the regulation of the hypothalamic–pituitary–adrenal axis.

### 3.5. Strains WL11 and WL17 Alleviate the Anxiety-Like Behavior, Improve Motor Coordination and Memory Cognition, and Regulate the Oxidative Stress and Inflammatory Factors in CFS Mice

**Figure 5:**
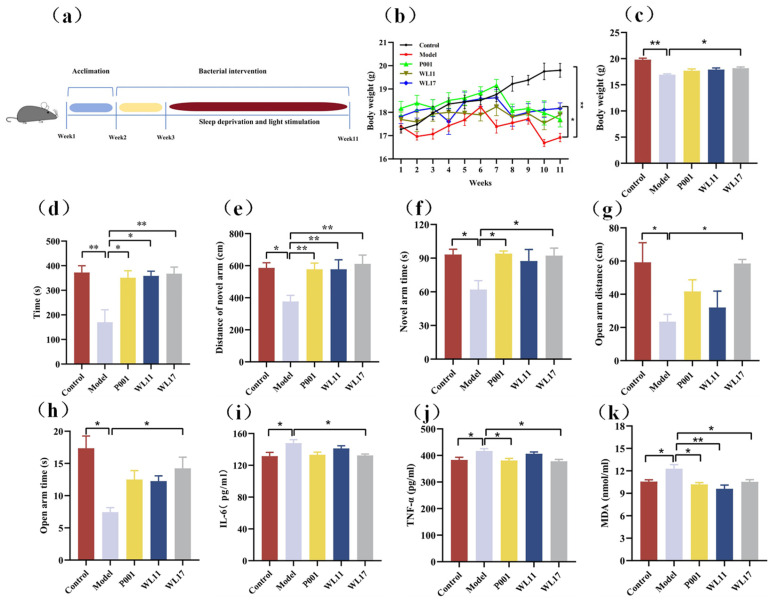
*Ls. rhamnosus* improved exercise capacity indicators, recognition memory, and anxiety-like behavior in CFS mice. Experimental design (**a**); body weight changes (**b**); and body weight at end (**c**); residence time of mice on rotating rod (**d**); distance of motion in novel arm (**e**); time spent in novel arms (**f**); distance of motion in novel arm (**g**); time spent in open arms (**h**); and effects of *Ls. rhamnosus* on expression of inflammatory cytokines IL-6 (**i**), TNF-α (**j**), and MDA in mouse serum (**k**). Strain P001 was from Wonderlab storage and was evaluated in parallel in all tests. Statistics: * *p* < 0.05, ** *p* < 0.01.

We used the methods of sleep deprivation and light stimulation for the generation of CFS model mice (Figure 5a). Based on the behavior and other changes in the CFS model mice and the control group of mice (Figure 5b–k), we concluded that the modeling process was successful. All the CFS mice with *Ls. rhamnosus* intake tended to recover their body weight, compared to the body weight loss of CFS model mice without *Ls. rhamnosus* intake (Figure 5b), but only that of strain WL17-intervened mice showed significant recovery of their body weight at the end of the experiments (Figure 5c). The rotating rod test was performed to evaluate the mice’s ability to exercise endurance and motor coordination. Compared with the control group of mice, the CFS model group of mice showed a significant reduction in the rotating rod time. All CFS model mice that went through interventions with the strains of *Ls. rhamnosus* significantly restored their locomotivity (Figure 5d), suggesting that the *Ls. rhamnosus* strains had good anti-fatigue properties. The Y-maze assay was utilized to investigate the potential effect of *Ls. rhamnosus* strains on improving learning and memory function impairment in CFS mice. As shown in Figure 5e,f, the CFS model group of mice had shorter distances of novel arms and less residence time in the novel arm, an indication that the learning and memory function of CFS model mice were damaged. Intervention with strain WL17 significantly restored the moving distance and time in the novel arm (Figure 5e,f), suggesting an improvement in the learning and memory function of CFS mice. The EPM tests showed that the anxiety-like behavior that appeared in the CFS model mice was significantly alleviated by strain WL17 treatment, as the distance and duration of open arm movement in CFS mice significantly increased (Figure 5g,h).

The concentration levels of the pro-inflammatory and oxidative stress cytokines TNF-α, IL-6, and MDA in the mouse serum were used to evaluate the effects of *Ls. rhamnosus* on the CFS mice. As shown in Figure 5i–k, compared with the control group, the level of IL-6, TNF-α, and MDA significantly increased in the CFS model group. The experimental group of mice intervened with strain WL17 had statistically lower levels of IL-6 and TNF-α, compared to those of the CFS model group mice (Figure 5i,j). All the tested strains reduced oxidative stress responses, as indicated by the reduction in MDA levels in the *Ls. rhamnosus*-treated mice (Figure 5k).

This study aimed to discover new resources of *Ls rhamnosus* strains, and two newly isolated strains, WL11 and WL17, were obtained for the alleviation of CFS and IBS syndromes. Metabolites such as SCFAs from strains WL11 and WL17 might partly be the molecules that mediated their probiotic functions. The ability to degrade multiple complex carbohydrates is one of the key genetic strategies to ensure successful colonization and survival of probiotic bacteria in the intestinal environment [50]; SCFAs are fermentative products from complex polysaccharides, and over 95% of SCFAs in the intestine are acetic acid, propionic acid, and butyric acid, with very little content of valeric acid and isovaleric acid [51]. Acetic acid serves as an energy substrate for peripheral tissues [52]. Clinical trials have also shown that acetate can have a positive effect on chronic stress and stress-induced intestinal permeability [53]. SCFAs can cross the blood–brain barrier and affect the production of brain neuropeptides [54]. These characteristics may contribute to the alleviation of memory impairment and anxiety-like behavior in CFS mice. Based on the research, strains WL11 and WL17 not only alleviate symptoms related to CFS but also alleviate visceral hypersensitivity and anxiety-like behavior in IBS. The multifunctionality doubles the health benefits of those probiotic strains and would provide more comprehensive health benefits and added value to probiotic products. However, the results from mouse models cannot be translated directly into human patients, because human patients are more complicated and many factors such as psychological and emotional states are not able to be mimicked in mouse models. Nevertheless, the new *Ls. rhamnosus* probiotics strains and their effectiveness on the alleviation of IBS and CFS are worthy of further evaluation in clinical trials in the future.

## 4. Conclusions

In this study, twenty-one strains of *Ls. rhamnosus* were isolated and characterized for their genomic and phenotypic features. Whole genome sequencing and comparative genomic analysis indicate that the pan-genome of *Ls. rhamnosus* is open and encodes multiple carbohydrate enzyme activities, which are consistent with their phenotypes. Strains WL11 and WL17 were selected for potential probiotics and subjected to evaluation with mouse models. The strains WL11 and WL17 effectively alleviate slow body weight gain, anxiety, poor memory, and cognitive impairment in CFS mouse models; reduce the expression of pro-inflammatory factors such as TNF-α and IL-6; and alleviate intestinal peristalsis, visceral hypersensitivity, and anxiety-like behavior in IBS model mice. Strain WL17 has more potential for alleviating symptoms related to IBS and CFS.

## Data Availability

The genomic data in this study were deposited in the National Microbiology Data Center with accession number NMDC10018728 (https://nmdc.cn/resource/genomics/genome/detail/NMDC10018728, accessed on 16 May 2024).

## Supplementary Materials

The following supporting information can be downloaded at https://www.mdpi.com/article/10.3390/microorganisms12061081/s1. (1) Figure S1: Genome annotation for antibiotic resistance sensitivity and prediction of virulence factors; (2) Table S1: Generation time and tolerances to acidity/alkalinity by 9 strains of *Ls. rhamnosus*.
