# Supplementary material for: Lacticaseibacillus rhamnosus Strains for Alleviation of Irritable Bowel Disease and Chronic Fatigue Syndrome
Source: Microorganisms. 2024 May 27;12(6):1081. doi: 10.3390/microorganisms12061081 (PMC11205684; doi:10.3390/microorganisms12061081)
Supplement: Supplementary file 1 [file microorganisms-12-01081-s001.zip › microorganisms-3002785-supplementary.pdf]

Lacticaseibacillus rhamnosus Strains for Alleviation of Irritable Bowel Disease and Chronic Fatigue Syndrome

Liang Zhang [1,†], Xue Ni [1,†], Minzhi Jiang [1], Mengxuan Du [1], Shuwen Zhang [1], He Jiang [1], Chang Liu [1,*] and Shuangjiang Liu [1,2,*]

[1] State Key Laboratory of Microbial Technology, Shandong University, Qingdao 266237, China; 202190900070@sdu.edu.cn (L.Z.); nixue1140317984@foxmail.com (X.N.); jiangminzhi2006@126.com (M.J.); dumengxuan2014@126.com (M.D.); 202000141061@mail.sdu.edu.cn (S.Z.); jiangh@sdu.edu.cn (H.J.); liu.c@sdu.edu.cn (C.L.)
[2] State Key Laboratory of Microbial Resources, Institute of Microbiology, Chinese Academy of Sciences, Beijing 100101, China
* Correspondence: liusj@im.ac.cn
† These authors contributed equally to this work.

(a)

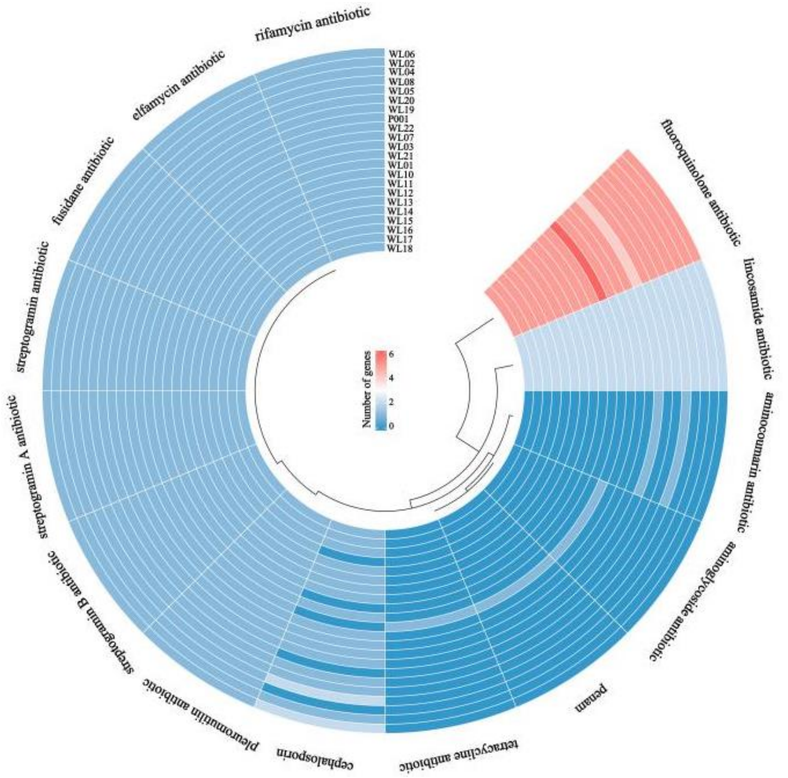

(b)

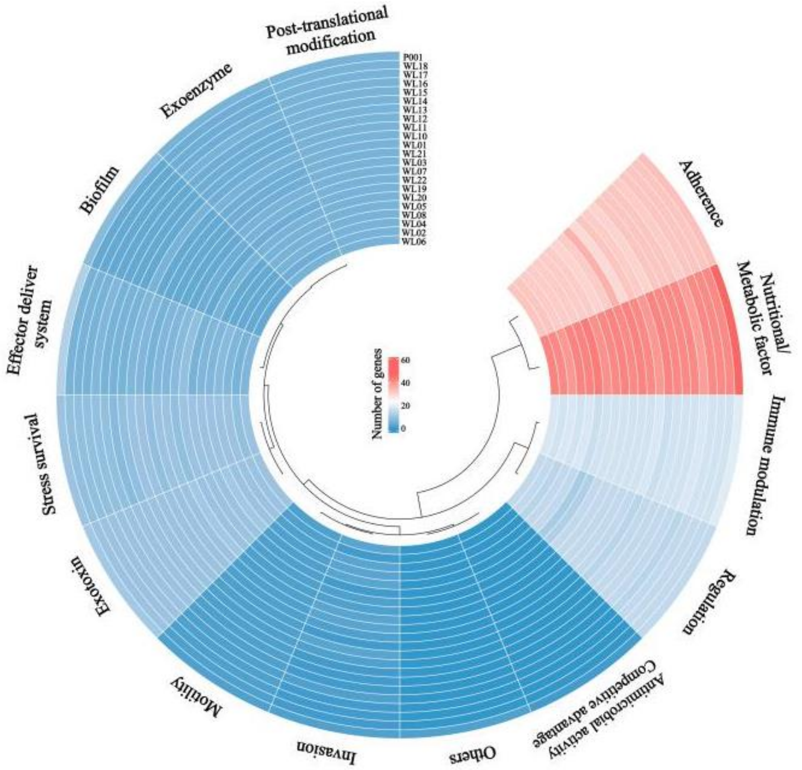

Supplementary Figure S1. Genome annotation for antibiotic resistance sensitivity (a) and prediction of virulence factors (b) in the genomes of *L. rhamnosus* strains. The *L. rhamnosus* strain P001 was analyzed in parallel for reference.

Supplementary Table S1 Generation time, and tolerances to acidity/alkalinity by 9 strains of *L. rhamnosus*

Strain	Generation time (min)	survival rate (%)				
		pH 3	pH 3.5	pH 4	pH 9	pH 10
P001	128.8±3.7	0	98.6±0.7	99.07±1.05	98.1±0.4	98.1±0.7
WL10	121.8±2.6	52.9±1.6	99.9±0.4	99.1±0.4	99.5±0.9	99.8±0.2
WL11	112.7±6.1	97.4±0.1	98.9±0.8	98.5±0.4	99.1±0.6	98.4±1.9
WL12	141.5±3.9	0	94.9±1.1	99.6±0.1	99.6±0.7	94.2±0.6
WL13	132.1±5.9	0	98.4±0.3	98.6±0.6	99.5±0.5	99.7±0.6
WL14	122.9±3.7	0	98±0.8	96.1±1.0	98.5±1.4	97.5±0.8
WL15	121.8±2.5	0	98.2±0.5	98.4±0.8	98.6±0.6	97.4±1.3
WL16	159.3±8.3	0	98.9±0.4	98.9±0.5	98.9±0.9	98.3±0.8
WL17	112.9±1	91.2±0.6	96.6±0.9	99.4±0.4	97.7±0.7	98.5±0.6
WL18	129.1±0.6	96.5±0.3	98.5±0.6	99.1±0.6	99.1±0.8	99.8±0.1
